# Assessing the efficacy of frankincense extract as a root canal irrigant against *Enterococcus faecalis*

**DOI:** 10.1371/journal.pone.0321458

**Published:** 2025-04-09

**Authors:** Rahaf A. Almohareb, Reem M. Barakat, Eltayeb E.M. Eid, Albandari Aldaws, Nourah Alhagbani, Reham Almubayi, Dhuha Alsuwaid, Fahda N. Algahtani

**Affiliations:** 1 Department of Clinical Dental Sciences, College of Dentistry, Princess Nourah Bint Abdulrahman University, Riyadh, Saudi Arabia; 2 Dental Clinics Department, King Abdullah bin Abdulaziz University Hospital, Princess Nourah Bint Abdulrahman University, Riyadh, Saudi Arabia; 3 Research Department, Health Science Research center, Princess Nourah bint Abdulrahman University, Riyadh, Saudi Arabia; 4 Research & Development Theme, Biotischen Industerial Inc., Riyadh, Saudi Arabia; 5 Dental Intern, College of Dentistry, Princess Nourah bint Abdulrahman University, Riyadh, Saudi Arabia; 6 Academic Researcher, Natural and Health Sciences Research Center, Princess Nourah bint Abdulrahman University, Riyadh, Saudi Arabia; 7 Department of Clinical Dental Sciences, College of Dentistry, Princess Nourah Bint Abdulrahman University, Riyadh, Saudi Arabia; A T Still University Missouri School of Dentistry & Oral Health, United States of America

## Abstract

Frankincense resin exhibits antibacterial potential against various microorganisms, but little is available on its effectiveness against dental root canal biofilm. This study aimed to assess its efficacy as a root canal irrigant against *Enterococcus faecalis* biofilm. A standard *E. faecalis* strain underwent antibacterial sensitivity testing with frankincense derived from *Boswellia sacra Flück* and *Boswellia frereana Birdw* trees. Frankincense, demonstrating inhibition of bacterial growth, was further evaluated as an irrigant. Root canals of 50 single-canalled human teeth were prepared, then contaminated with *E. faecalis* and placed into three groups: Group A was irrigated with saline (negative control), Group B was irrigated with 5.25% sodium hypochlorite (NaClO), and Group C was irrigated with frankincense. Microbial sampling pre- and post-irrigation was conducted under aseptic conditions. Colony count reduction percentages were calculated, and the data was analyzed using one-way analyses of variance followed by Tukey’s post-hoc test (significance level set at 5%). The antibacterial susceptibility test revealed that only *Boswellia sacra Flück* frankincense was effective against *E. faecalis*. Both NaClO and frankincense significantly reduced colony counts compared to saline (*p* <  0.0001), with no difference between frankincense and NaClO irrigation. Therefore, root canal irrigation with *B. sacra* frankincense proved as effective against *E. faecalis* biofilm as NaClO. Further exploration of its potential as a root canal irrigant is recommended.

## Introduction

Apical periodontitis is a prevalent oral disease that can lead to tooth loss and ultimately affect the quality of life [1]. The primary cause of this disease is the endodontic biofilm [2]. To effectively treat root canal infections and resolve apical periodontitis, it is essential to successfully eliminate this biofilm. [2]. The key to this process is the irrigation protocol, which aims to eradicate the biofilm chemically and/or mechanically [3].

The endodontic biofilm harbors various bacteria. One prominent bacterium species is *Enterococcus faecalis*, which is associated with root canal treatment failures [4]. These Gram-positive cocci are commonly found in both the intestines and oral cavity. They possess the ability to infect dental pulp tissue and form resilient biofilms capable of enduring the harsh conditions of root canal treatment [5,6].

To date, the root canal irrigant of choice has been sodium hypochlorite (NaClO) [3,7,8]. It has effective antibacterial properties due to its high pH, reaching 11 [9]. Besides its antimicrobial properties, NaClO can dissolve the pulpal tissue and organic dentin components [10,11]. Furthermore, it can partially neutralize any antigenic or microbial substance remaining in the root canal space [12]. Increasing the NaClO concentration and temperature can greatly enhance its effectiveness and tissue-dissolving capacity [13,14]. However, NaClO is highly cytotoxic, and its accidental extrusion into the periapical tissue can result in adverse effects ranging in severity from transient to severe pain and different levels of localized tissue necrosis, hematoma, edema, and neurological damage.

Over the years, plants have been used for medical purposes as part of commercial medication or in herbal treatments. For oral infections, plant-based phytochemicals obtained from extracts such as *Carissa bispinosa Desf.* have shown promising antibacterial activity against oral pathogens [15]. The use of nanoparticles has been promoted to enhance the antibacterial properties of dental materials. However, a major concern with incorporating nanoparticles into these materials is the potential toxicity to patient tissues when exposed for extended periods [16].

The *Burseraceae* family consists of plants that produce resin and is particularly famous for two genera: *Commiphora,* which produces myrrh, and *Boswellia*, which produces frankincense [17,18]. The latter, a high-quality oleo-gum resin, is a popular material used by the inhabitants of the Arabian Peninsula and North Africa. Frankincense resin is obtained through incisions made in the *Boswellia* tree trunk. The genus *Boswellia* contains 25 species, which mainly inhabit Northern Africa: *Boswellia carterii Birdw* and *Boswellia frereana Birdw (B. frereana)*, Ethiopia (*Boswellia papyrifera Hochst* and *Boswellia rivae Engl*), and Eritrea (*Boswellia neglecta S. Moore*), in addition to *Boswellia serrata Roxb. (B.serrata)* in India, *Boswellia sacra Flück (B.sacra)* in the Arabian Peninsula [19]. The word “frankincense” is derived from an old French term meaning “pure incense” or “pure and noble high-quality incense.” Many scientists have sought to identify its medical effects [20]. Consequently, several published studies have reported the anticancer, anti-inflammatory, immunomodulatory, antimicrobial, and antiviral activities of frankincense obtained from several *B.* species [20,21].

A recent study highlighted the effects of different frankincense compounds on oral infection and inflammation [22]. The resin extracted from *B. sacra* exhibited antimicrobial activity against various microorganisms, including *Staphylococcus aureus, Escherichia coli*, *Proteus vulgaris*, and the fungal pathogen *Candida albicans* [23]. Furthermore, El-Nagerabi et al. found that oil extracted from the *B*. *sacra* showed remarkable activity against *Aspergillus parasiticus* and *Aspergillus flavus* growth [24]. This effect is related to the essential oils found in frankincense, which contain terpenoids such as α- and β-pinene, limonene, and linalool, which are known for their antibacterial properties [25,26]. The essential oils and antibacterial efficacy have been reported to differ among frankincense harvested from different locations [26,27].

Boswellic acids, a class of pentacyclic triterpenic acids, are identified as the active compounds in frankincense. Research has highlighted their significant role in mitigating chronic inflammatory diseases [28,29]. These compounds were found to endow frankincense with notable anti-cancer properties [30,31], while having no adverse effect differentiated and stem cell viability [32,33]. Further studies have indicated that different *Boswellia* species vary in their boswellic acid content, which influences their pharmacological activities [34].

A novel therapeutic strategy involving mesoporous bioactive glass nanoparticles loaded with *B. sacra* extract showed promising antibacterial activity and improved tissue healing [35]. A recent study reported that *B.serrata* extracts inhibited *Porphyromonas gingivalis* biofilm formation and significantly decreased biofilm biomass [36].

Therefore, this study aimed to compare the efficacy of frankincense from *Boswellia* tree species as a root canal irrigant against *E. faecalis* biofilm to NaClO. Its null hypothesis was that the antibacterial effects would not differ between NaClO and frankincense.

## Materials and methods

This ex vivo study was granted ethical approval by the Internal Review Board of Princess Nourah bint Abdulrahman University (IRB no. 23-0690).

### Preparation of the frankincense and bacterial species

A standard *E. faecalis* strain (American Type Culture Collection: 29212) [37], was cultured on bile esculin agar in a 37°C incubator for 24 hours. After 24 hours, a sterile swab was used to collect a single *E. faecalis* colony and suspend it in 3.0 mL of sterile brain heart infusion (BHI) broth, which was mixed using a vortex until homogenous. Then, the optical density of the suspension was measured in McFarland units using a DensiCHEK Plus density meter (bioMérieux, Inc., Durham, NC, USA).

**Fig 1:**
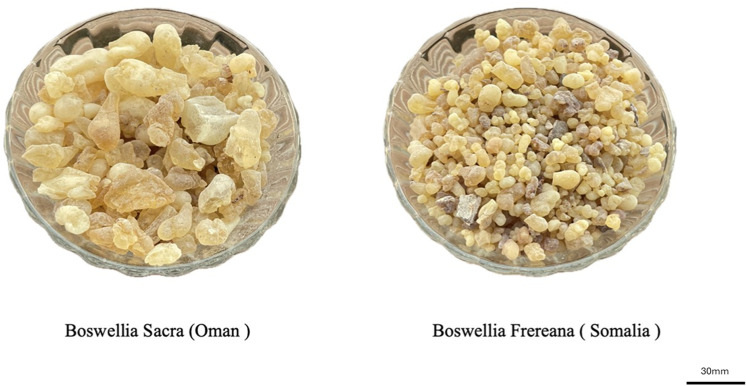
Frankincense gum resin obtained from *B.sacra* and *B.* Frerena.

Frankincense gum resin was obtained from two *Boswellia* species: *B. frereana* (collected in Somalia) and *B. sacra* (collected in Oman). They were crystalline, with colors ranging from white (*B. sacra)* to yellow (*B. frereana)*; (Fig 1).

Frankincense was prepared by grinding the resin using a pestle and mortar, then soaking the resulting powder in 95% ethanol at a ratio of 625 mg per 1 mL for 72 hours at room temperature to preserve all ingredients. The mixture was filtered with Whatman No. 1 filter paper, then placed in a rotary evaporator. The concentrate was further incubated at 37 °C for two days to allow the solvent to evaporate. The final extract was stored in an aseptic, airtight container. An irrigation solution was prepared using the extract at a concentration of 6.25 mg/mL.

### Antibacterial susceptibility test

The antimicrobial activity of the two frankincense extracts was examined using the agar-well diffusion method. Two bile esculin agar plates were prepared, one for the *B. frereana* extract and the other for the *B. sacra* extract. Each plate had two 4 mm wells cut into it using sterile tips, which were filled with 50 µL of the respective frankincense solution using a sterile pipette. Then, these plates were incubated at 37°C for 24 hours. Following incubation, the inhibition zones around the wells were observed to assess antimicrobial effectiveness. The frankincense extract showing effective inhibition zones was selected for performing the root canal irrigation test.

### Ultra performance liquid chromatography (UPLC)

Liquid chromatography provides an efficient approach for the precise quantification and analysis of the characteristic acidic compounds found in the *B. sacra*, enhancing accuracy, selectivity, and sustainability in natural product research. To characterize boswellic acid, standard solutions of α-Boswellic acid (CAS No.: 471-66-9) and β-Boswellic acid (CAS No.: 631-69-6) with purities of 98.40% and 99.89%, respectively, purchased from (MedChemExpress, NJ, USA) were injected into the UPLC system. Liquid chromatography was performed using ACQUITY™ UPLC system (Waters Corp., Milford, MA, USA) with an autosampler set at 15 °C. Separation was conducted on a BEH Amide column (1.7 µm, 2.1 x 100 mm, Waters Corp., Milford, MA, USA) at 40 °C. The mobile phase consisted of 85% acetonitrile and 15% 0.1 M potassium dihydrogen orthophosphate (pH 2.5, adjusted with orthophosphoric acid), delivered at a flow rate of 0.40 mL/min.

Accurately weighed 1 mg of α- and β-boswellic acids was dissolved in 1 mL chloroform, vortexed for 3 minutes, and diluted to 100 µg/mL. A 0.2 µL volume of each standard was injected into the UPLC system to determine the exact elution profile of boswellic acid under established conditions. A standard curve was generated at concentrations of 10, 20, 30, 40, and 50 µg/mL.

A 174 mg sample of the crude ethanolic frankincense extract that showed effective inhibition zones, was accurately weighed, dissolved in 1 mL chloroform, vortexed for 3 minutes, and diluted 10-fold. A 0.2 µL volume of the prepared extract was injected into the UPLC system

**Fig 2:**
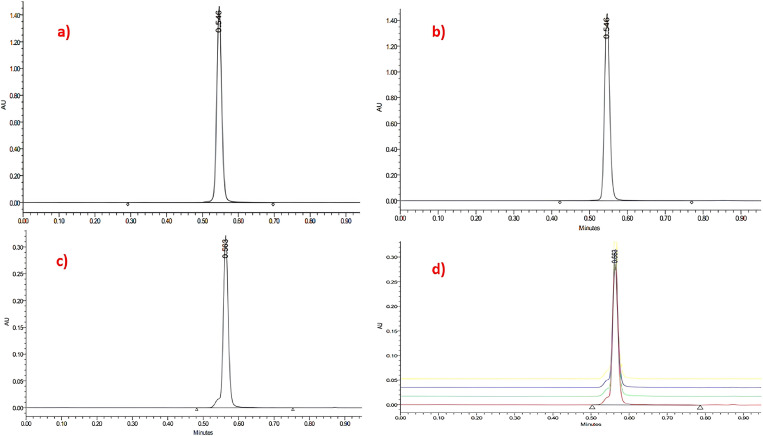
Ultra performance liquid chromatography results show in images (A), (B) the distinct peaks for the standard α-boswellic acid and β-**boswellic acid, while image (C) shows the peaks for the experimental ***B.sacra*** extract.** The peak at 0.546 retention time in all images indicates the *B.sacra* extract content of the boswellic acids.

The total run time of less than one minute demonstrated the method’s sensitivity and selectivity, as each isomer displayed a distinct peak without interference. The standard curve for boswellic acid was linear over the range of 10–50 µg/mL (R² =  0.9981), and no interfering peaks were observed, underscoring the method’s selectivity (Fig 2).

Using the standard curve equation*: Υ = 17976X − 1531.9*, extraction recovery was calculated. Quantification was performed in triplicate, based on the mean peak height. The 174 mg crude extract contained 0.102 mg of pure boswellic acid, equating to 0.586 mg per gram of crude extract. Thus, from 25 g of Boswellia, a recovery of 58.62% pure boswellic acid is expected.

### Antibacterial susceptibility test

The antimicrobial activity of the two frankincense extracts was examined using the agar-well diffusion method. Two bile esculin agar plates were prepared, one for the *B. frereana* extract and the other for the *B. sacra* extract. Each plate had two 4 mm wells cut into it using sterile tips, which were filled with 50 µL of the respective frankincense solution using a sterile pipette. Then, these plates were incubated at 37°C for 24 hours. Following incubation, the inhibition zones around the wells were observed to assess antimicrobial effectiveness. The frankincense extract showing effective inhibition zones was selected for performing the root canal irrigation test.

### Tooth preparation

The sample size required for this study was estimated using the G * Power 3.1 software (Heinrich-Heine-Universität, Düsseldorf, Germany), considering a power of 90%, a type 1 error probability (*α*) of 0.05, and effect size (*f*) =  0.5. The estimated sample size was 54 teeth.

A total of 56 teeth, extracted for reasons unrelated to this study, were used after obtaining written patient consent. Teeth were stored in saline until used. The exclusion criteria included teeth with curved canals, external and internal resorption, double canals, and calcified canals. The crowns were removed, and root lengths were standardized at 16 mm. Rotary nickel-titanium instruments (ProTaper Universal, Dentsply Sirona, Charlotte, NC, USA) were used following the manufacturer’s instructions. The instruments were used with a 6:1 reduction contra-angle connected to a rotary electric Endo motor (X-smart, Dentsply Sirona, Charlotte, NC, USA). Canals were irrigated with 5 mL of 5.25% NaClO (Pharma Vitality, Riyadh, Saudi Arabia) between each file. A final rinse alternated between 5 mL of 5.25% NaClO with 3 mL of 17% ethylenediaminetetraacetic acid for one minute and 5 mL of saline [3]. The teeth were autoclaved at 126°C for 20 minutes.

A standard *E. faecalis* strain (American Type Culture Collection: 29212) was cultured in BHI broth at 37°C for 24 hours. Each tooth was immersed in 900 µ L of BHI broth and 300 µ L of BHI broth containing bacteria and incubated at 37°C in a shaking incubator at 180 rpm for two weeks. The BHI broth was renewed every two days by discarding 300 µ L and replacing it with 300 µ L of fresh BHI broth under sterile conditions.

**Fig 3:**
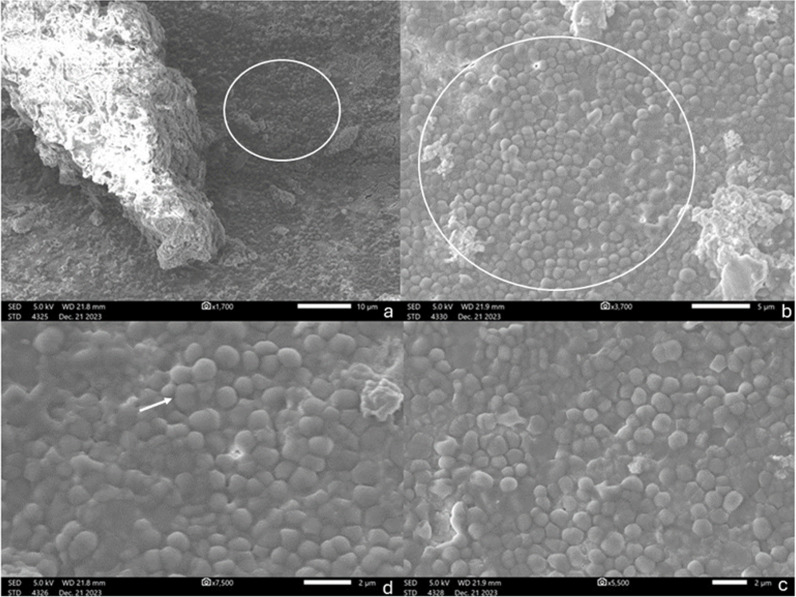
SEM images of canal walls exhibiting the biofilm formation by E. faecalis. Image (a) provides a comprehensive view of the biofilm, with a highlighted area encircled in white that is magnified in images (b) and (c), revealing the biofilm structure. In image (d), an arrow indicates the distinctive oval-shaped morphology of these resilient Gram-positive cocci, showcasing their characteristic arrangement and adaptive surface features.

Next, two teeth were randomly selected and sectioned using a Needle Diamond Bur 0.10 mm (Strauss Diamond, Palm Coast, FL, USA). Then, they were coated with platinum via an auto-fine coater (JEC-3000FC; Jeol, Tokyo, Japan) and examined under an SEM (JSM-IT500HR; Jeol, Tokyo, Japan) with high vacuum conditions at 5 kV with a probe current of 35 A to confirm the existence of *E. faecalis* biofilms (Fig 3).

### Root canal microbial sampling

Strict aseptic conditions were maintained during microbial sampling, which was performed before and after irrigation by a single operator. The first sampling was performed for all teeth by filling the canal with sterile 0.9% normal saline (NS) and then circumferentially filing it with a size 40 H-file (MANI, Inc., Tochigi, Japan) inserted 1 mm short of the working length. The canal contents were absorbed into ProTaper Universal F3 paper points left in the canals for 60 seconds before being transferred into test tubes containing 1.0 mL of NS. The contents of each canal were serially diluted and plated on BHI agar plates. For the second microbial sampling, the teeth were randomly divided into three groups. Teeth in Group 1 served as negative control and were irrigated with 10 mL of saline (*n* =  18). Teeth in Group 2 were irrigated with 10 mL of 5.25% NaClO (*n* =  18). Group 3 represented the experimental group, and teeth were irrigated with 10 mL of the *B. sacra* frankincense solution (*n* =  18). The irrigant was inserted into the canal using a 30-gauge side-vented irrigation needle placed 1 mm short of the working length. The irrigant was injected at a flow rate of approximately 10 mL per minute, simulating the clinical situation, and the irrigant was left in the canal for one minute.

An operator blinded to the groups performed the serial dilution and plating on BHI agar plates in steps identical to those used for the first sampling. Following incubation for 24 hours, the colonies formed on the agar plates were counted on magnified images of the plates. The percentage reduction in colony forming units (CFU) from before to after irrigation was calculated using the following formula:


$$\frac{\text{CFU pre-irrigation – CFU post-irrigation}}{\text{CFU pre-irrigation}}\;\times \;100$$

Five random teeth from Group 3 were selected for SEM analysis (JSM-IT500HR; Jeol, Tokyo, Japan). Following sectioning, the teeth underwent a dehydration process at room temperature using an increasing ethanol gradient (70%, 80%, 90%, and 100%), immersing them in the water-ethanol mixture for 24 hours at each step. Following dehydration, the specimens were further dried in a desiccator for two hours. Finally, they were coated with platinum using an auto-fine coater before examination under the SEM.

### Statistical analysis

The data were analyzed using the SPSS software (version 22; SPSS Inc., Chicago, IL, USA) using one-way analyses of variance followed by Tukey’s post hoc test. A *p* <  0.05 was considered statistically significant.

## Results

### Antibacterial susceptibility test

**Fig 4:**
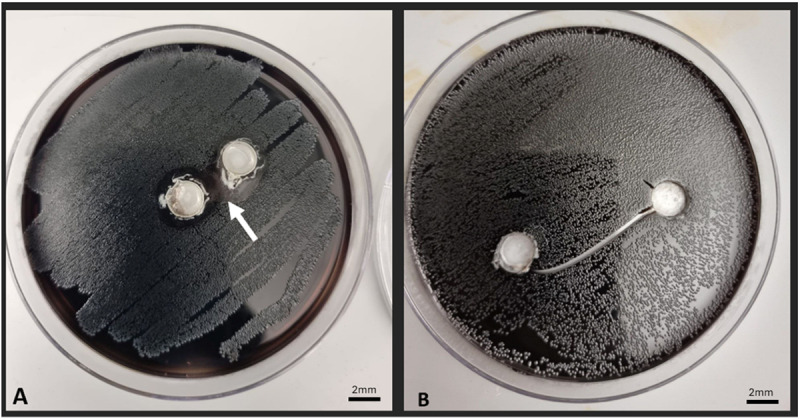
The agar-well diffusion method shows (A) *Enterococcus faecalis* growth inhibition with frankincense from *Boswellia sacra* and (B) the absence of inhibition zones with frankincense from *Boswellia frereana.*

The antibacterial susceptibility test showed that only frankincense from *B. sacra* had effective antibacterial activity against *E. faecalis*, as evidenced by clear zones of growth inhibition measuring 1.5–2 mm wide (Fig 4). Consequently, this specific type of frankincense was selected as the root canal irrigant for the experimental group.

### Root canal microbial sampling

**Fig 5:**
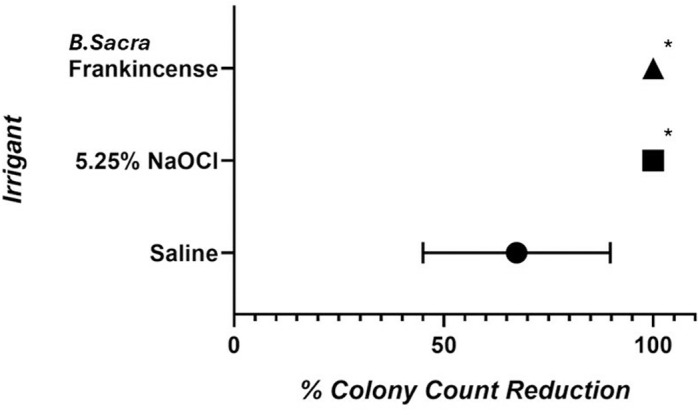
The percentage reduction in *E. faecalis* colony counts in the three groups.

**Fig 6:**
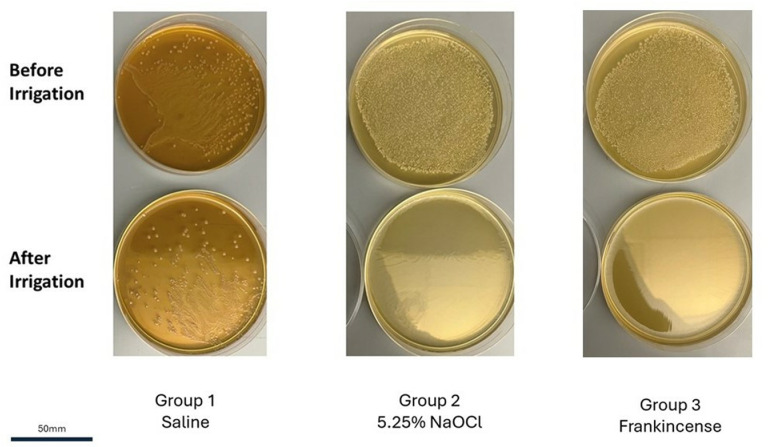
Images showing bacterial colony growth on agar plates before and after irrigation with the different solutions.

A significant 100% reduction in bacterial count was observed with both NaClO and frankincense from *B. sacra.* This reduction was statistically significant when compared to the control saline group (p < 0.0001), where the average reduction in bacterial count was 68.5% (Fig 5). The log10 reduction in CFU/mL counts showed significant differences among the irrigant groups (Table 1). Saline demonstrated the lowest antibacterial activity with a mean reduction of 0.571 ± 0.247. Both NaClO and *B. sacra* frankincense exhibited significantly higher reductions (2.993 ± 0.329 and 3.029 ± 0.130 respectively), indicating superior antibacterial efficacy compared to saline. The effect size (𝜂^2^ =  0.95) demonstrated that the irrigant accounts for a substantial proportion of the variance, indicating a large effect. The reduction in *E. faecalis* bacterial count did not differ significantly between *B. sacra* frankincense and NaClO irrigation (*p* =  1.000; Fig 6). These results highlight the antibacterial efficacy of both NaClO and frankincense from *B. sacra* against *E. faecalis*.

**Table 1:** Mean log₁₀ values for the reduction in colony forming units pre and post irrigation with the different irrigants.

Irrigant Group	N	Mean	Std. Deviation	95% Confidence Interval	
				Lower Bound	Upper Bound
Saline	18	0.571^b^	0.247	0.448	0.693
5.25% Sodium Hypochlorite	18	2.993^a^	0.329	2.830	3.157
B.Sacra Frankincence	18	3.029^a^	0.130	2.965	3.094

Different letters indicate significant difference with p < 0.0001.

* *p* < 0.0001 compared to the saline control.

**Fig 7:**
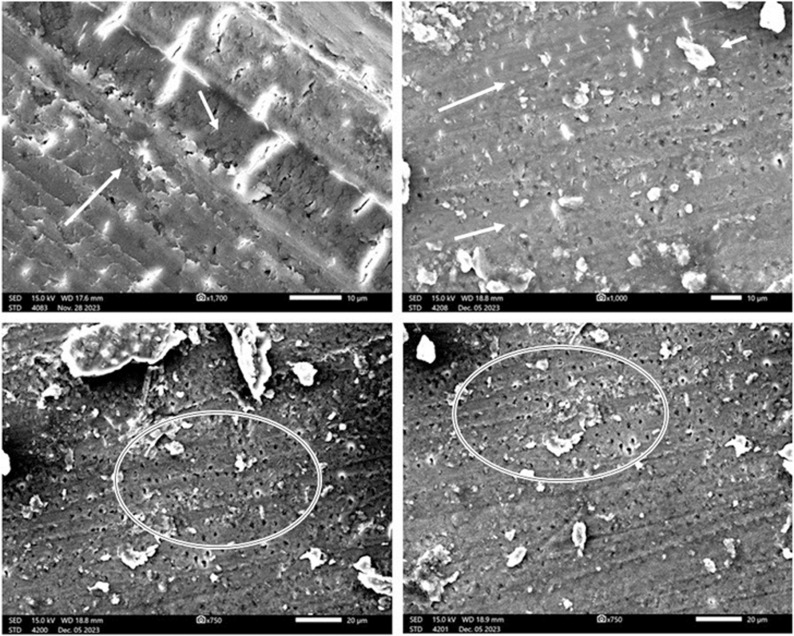
Scanning electron microscope images of the canal walls post-irrigation with *B. sacra* frankincense demonstrate absence of bacterial biofilm, with the visible openings of the dentinal tubules (encircled in white). Other areas exhibited the presence of a smear layer and debris, as indicated by the white arrows.

No bacterial biofilm was evident on the canal walls of the teeth irrigated with *B. sacra* frankincense in the scanning electron microscopy (SEM) images, with a smear layer observed in some locations, probably due to the filing of the canal walls (Fig 7).

## Discussion

Irrigation is a crucial part of successful endodontic treatment. Irrigating solutions facilitate the removal of microorganisms, necrotic and inflamed tissue, and dentine debris [38]. They also reduce friction between the instrument and dentine and improve the file-cutting effectiveness [3]. An optimal root canal irrigation procedure should eliminate bacterial biofilm and the smear layer, ensuring thorough disinfection of all aspects of the root canal.

NaOCI is a very effective disinfectant with desirable characteristics such as dissolving tissues, proteolytic activity, and bactericidal effects on endodontic biofilms [39], making it the preferred irrigation solution. Because of its high pH (between 11 and 12), NaClO is especially harmful to vital tissues and cytotoxic. NaClO accidents during root canal treatment may be rare, but if they do occur, they can result in serious patient morbidity and suffering. The presence of certain pathological and iatrogenic conditions, such as root canal perforation and external root resorption, can exacerbate this issue [40]. The often-seen clinical consequences of a NaClO accident include discomfort, ecchymosis, ulceration, edema, neurologic damage (anesthesia and paraesthesia), chemical burns and necrosis, and, at times, respiratory compromise [41].

The antibiofilm efficacy of NaClO is influenced by two key factors: the duration of contact between the solution and biofilm and its concentration. A 1% NaClO solution failed to completely eradicate all *E. faecalis* biofilm, indicating that higher concentrations should be used (2.5% and 5.25%), which are associated with increased cytotoxicity [42].

*E. faecalis* has been associated with failed root canal treatment due to its ability to endure challenging conditions [43]. Multiple studies have demonstrated that *E. faecalis* exhibits a high capacity to form biofilms on human dentin after 72 hours. Furthermore, the bacteria present in mature biofilms are significantly more resistant to NaClO. Irrigation with 2.5% or 5.25% NaClO within 10 minutes is necessary to effectively eradicate such biofilm [44].

The present study investigated the efficacy of frankincense derived from different *Boswellia* tree species as a root canal irrigant against E. faecalis biofilm. One of the major advantages of frankincense is its biocompatibility with oral tissues [35,45,46]. It has been traditionally used as an oral rinse and in the management of cough and asthma [22]. Additionally, previous studies have demonstrated that frankincense does not adversely affect the viability of heart cells or dental pulp stem cells [32,33]. The current findings demonstrated that frankincense from *B. sacra* was as effective as 5.25% NaClO against *E. faecalis* biofilm. Therefore, the null hypothesis was accepted. This finding aligns with a recent study examining the effectiveness of *B. sacra* extract as an intracanal medicament compared to calcium hydroxide against *E. faecalis* biofilm. In that study, confocal laser scanning microscopy revealed that *B. sacra* penetrated deeper into the dentinal tubules and exhibited superior effectiveness within a three-day application period. Even after seven days, calcium hydroxide did not surpass the performance of *B. sacra* [47]*.* Other studies also found that frankincense from *B. serrata* extracts notably reduced the amount of *P. gingivalis* biofilm compared to conventional antibiotics such as penicillin and streptomycin [36]. Therefore, it is recommended for treating periodontitis [46]. However, this effect did not extend to *Fusobacterium nucleatum* biofilms, highlighting that frankincense’s ability to inhibit bacterial growth depends on the type of bacteria [20]. Future studies, incorporating quantitative methods such as minimum inhibitory concentration (MIC) determination or microdilution assays would provide more precise and standardized insights into the antimicrobial effectiveness of the extract. Further studies could also investigate the effect of varying irrigation volume to better understand its impact on the antimicrobial efficacy.

Frankincense was reported to show superior efficacy against Gram-positive bacteria than Gram-negative bacteria [35], consistent with our current observation, as *E. faecalis* is Gram-positive. A separate study evaluated the antibacterial potential of extracts and essential oils from *B. sacra* and *Boswellia papyrifera Hochst* trees against various bacteria. Its results also revealed that the essential oil from *B. sacra* created the most significant zone of inhibition against *E. faecalis*, surpassing the effectiveness of ticarcycline [48].

Multiple studies have explored the therapeutic potential of frankincense derived from different *Boswellia* species, highlighting its antibacterial, anticancer, anti-inflammatory, and antiviral abilities [49]. Frankincense from the *B. serrata* species found in India effectively prevented the formation of *Streptococcus mutans* biofilm [50]. It proved effective in treating gingivitis, leading to a significant decrease in inflammatory indices. Notably, no significant difference was observed between using the powder or extract form of frankincense [45].

Two varieties of frankincense: *B. sacra* and *B. frereana* were explored in this study. However, only the former created an inhibition zone in the *E. faecalis* susceptibility test. This inhibition zone may appear smaller than what has been reported for other plant-based antibacterial agents, such as Matricaria chamomilla, which are proposed for root canal treatment [51]. However, the thickness of the agar media used in the bacterial susceptibility test can influence the size of the inhibition zone; thinner media tend to produce larger zones [52].

Hasson et al. compared the antibacterial properties of *B. sacra* and *B. frereana* extracts against various bacteria (*S. aureus, Pseudomonas aeruginosa, and Streptococcus pneumoniae*) and concluded that methanol extracts of *B. sacra* from Oman had superior antibacterial activity to those of *B. frereana* from Somalia. They concluded that the antibacterial effect depends on the tree from which the frankincense is obtained and the solvent used for extraction [20]. In addition to water extracts, they used methanol extracts, like in other studies [48]. Samani et al. used a different extraction technique, which involved soaking finely ground frankincense oleo-gum in 95% ethanol for 48 hours, followed by filtration [45], which is similar to the technique used in our study.

Another explanation could be the difference in the *Boswellia* resin content of boswellic, lupeolic, and pentacyclic triterpenic acids (PTAs). One study found that *B. sacra* resins are particularly rich in PTAs, whereas these acids were not found in *B. frereana* resins [53]*.*

Anti-bacterial efficacy of frankincense was found to be related to the boswellic acids found in its gum resin. Acetyl-11-keto-b boswellic acid in particular is effective against Gram positive bacteria. It disrupts the bacterial cell membrane’s permeability causing the leaking of its cytosolic components [54–56]. However, the susceptibility of bacterial species to boswellic acids varies, for example, Gram negative bacteria showed resistance due to their outer membrane providing protection against hydrophobic compounds [55].

In the present study, a rotary evaporator, which is well-known for its effectiveness in eliminating volatile solvents such as ethanol was used to prepare the Frankincense extracts [57]. However, 20% ethanol was used to prepare the final irrigation solution with no additional purification processes. This residual ethanol may have influenced the anti-bacterial activity observed.

The quest for herbal alternatives to the gold standard, NaClO, in root canal irrigation has been scrutinized. Systematic studies have shown that many alternatives not only exhibit lower antibacterial efficacy than NaClO but also cannot dissolve organic tissue [58,59] or effectively remove the organic and inorganic components of the smear layer [60]. Whether to remove the smear layer remains contentious. Some argue for its preservation to impede further microbial infiltration [61], while others argue that its impact on clinical treatment outcomes is insignificant. However, there is a notable scarcity of clinical studies examining its role [62].

Recently, activated irrigation devices have been proposed to improve antibacterial efficacy while using lower NaClO concentrations [63]. However, these devices are costly, and their adjunct use can be time-consuming. Therefore, there is a keen interest in thoroughly exploring the potential of herbal agents to develop more efficient irrigation solutions. That said, there may be other practical challenges associated with using frankincense as a root canal irrigant. For one, potential costs and material availability and variability. The chemical composition of frankincense varies according to multiple factors, one of which is harvest conditions, leading to inconsistent extract quality. Not to mention the depletion of the Boswellia species ecosystem and their overharvesting. Better standardized extraction processes would be necessary to ensure that the sufficient amount of boswellic acids is yielded and that certain regulatory requirements such as residual solvent limits and purity standards are met. This may prove to be complex, challenging the scaling up of the process to an industrial level. For this reason, further research is required to determine the clinical applicability of this material.

Although frankincense has demonstrated potent antibacterial efficacy against a major endodontic pathogen, it is crucial to further investigate its additional properties related to irrigation. Key areas of focus should include its wetting ability, effectiveness in smear layer removal, interactions with other materials, and its impact on dentin strength. While the methodology used in our study replicated the clinical setting regarding irrigation protocol and contact with canal walls, it only targeted a single-bacteria biofilm, while the endodontic microbiota is multi-species. Therefore, further investigation of the efficacy of frankincense against the multi-species endodontic biofilm using methodologies that allow the visualization of the biofilm structure, such as confocal microscopy, is recommended.

## Conclusions

This study provides initial evidence of the effectiveness of frankincense from *B. sacra* as a root canal irrigant. It showed similar effectiveness to NaClO in eliminating E. faecalis biofilm. However, to fully understand its potential for clinical use, more research is needed. Further investigations are necessary to clarify its complete therapeutic benefits for application in dental practice including its clinical efficacy and long-term stability.

## Supporting information

S1 File Supplementary file F1. (XLSX)
